# Borderline ovarian tumours in Vaud, Switzerland: incidence, survival and second neoplasms

**DOI:** 10.1038/sj.bjc.6690002

**Published:** 1999-09-24

**Authors:** F Levi, C La Vecchia, L Randimbison, V-C Te

**Affiliations:** Registre Vaudois des Tumeurs, Institut Universitaire de Médecine Sociale et Préventive, Falaises 1, Lausanne, 1011, Switzerland; Istituto di Ricerche Farmacologiche ‘Mario Negri’Via Eritrea 62, Milan, 20133, Italy

**Keywords:** ovarian tumours, low malignant potential tumours, borderline tumours, serous tumours, mucinous tumours, incidence, time trends, cancer registration, survival, second primary

## Abstract

Between 1976 and 1996, 176 borderline ovarian tumours were registered in the Cancer Registry of the Swiss canton of Vaud, corresponding to an age-adjusted incidence (world standard) of 2.7 in 100 000. Incidence rose from 1.7 per 100 000 during 1976–81 to 2.7 per 100 000 during 1987–91, and then levelled off; 58% of cases were serous and 41% mucinous. Relative survival was 94% at 10 years; 18 second neoplasms were observed, compared with 10.3 expected, and there was a significant excess of invasive ovarian cancers (four observed, including three synchronous, compared with 0.4 expected). © 1999 Cancer Research Campaign


*British Journal of Cancer* (1999) **79**(1), 4–6
© 1999 Cancer Research Campaign

### Borderline ovarian tumours in Vaud, Switzerland: incidence, survival and second neoplasms

**F Levi[1], C La Vecchia[2], L Randimbison[1] and V-C Te[1]**

[1]Registre Vaudois des Tumeurs, Institut Universitaire de Médecine Sociale et Préventive, Centre Hospitalier Universitaire Vaudois, Falaises 1, 1011 Lausanne, Switzerland; [2]Istituto di Ricerche Farmacologiche 'Mario Negri', Via Eritrea 62, 20157 Milano, and Istituto di Statistica Medica e Biometria, Università degli Studi di Milano, Via Venezian 1, 20133 Milan, Italy

**Summary** Between 1976 and 1996, 176 borderline ovarian tumours were registered in the Cancer Registry of the Swiss canton of Vaud, corresponding to an age-adjusted incidence (world standard) of 2.7 in 100 000. Incidence rose from 1.7 per 100 000 during 1976–81 to 2.7 per 100 000 during 1987–91, and then levelled off; 58% of cases were serous and 41% mucinous. Relative survival was 94% at 10 years; 18 second neoplasms were observed, compared with 10.3 expected, and there was a significant excess of invasive ovarian cancers (four observed, including three synchronous, compared with 0.4 expected).

**Keywords:** ovarian tumours; low malignant potential tumours; borderline tumours; serous tumours; mucinous tumours; incidence; time trends; cancer registration; survival; second primary

Borderline ovarian tumours are epithelial ovarian cancers of low malignant potential, characterized by limited invasiveness, favourable prognosis and relatively early age at onset (Hart, 1977; Scully, 1982). Most information comes from clinical series (Barnhill et al, 1985; Nakashima et al, 1990; Rice et al, 1990; Leake et al, 1992; Kærn et al, 1993; Kennedy and Hart, 1996). Among the few published epidemiological series, the Western Washington Cancer Surveillance System (Harlow et al, 1987) showed a rising incidence between 1975 and 1983, from 0.7 to 3.9 per 100 000, a more modest rise with age compared with invasive neoplasms, and a 93% 5-year survival; 90 out of 168 (54%) cases were serous and 78 (46%) mucinous. In a clinical series of 370 patients (median age 53 years) treated between 1970 and 1982 at the Norwegian Radium Hospital (Kærn et al, 1993), 5-year survival was over 90% for both mucinous (48% of cases) and serous (47%) neoplasms. A total of 1197 borderline ovarian tumours were reported to the Finnish Cancer Registry between 1973 and 1992 (Auranen et al, 1996), corresponding to an age-standardized incidence of 1.8 per 100 000. Mean age was 52 years, and the incidence did not increase over age 35. Data from the Cancer Registry of Norway over the period 1954–93 showed an upward trend in incidence, reaching an age-adjusted rate of 4.8 per 100 000 in 1989–93. The incidence rose up to age 55, and then levelled off, and over 90% of neoplasms were localized (Bjorge et al, 1997). In Israel, the age-adjusted rate approached 2 per 100 000 in the early 1990s among Jews, representing about 10% of all ovarian neoplasms (Iscovich et al, 1998).

To provide further information on the issue, we examined incidence, survival and the occurrence of second neoplasms in borderline ovarian cancers diagnosed in the Cancer Registry of the Swiss Canton of Vaud.

*Received 1 April 1998*
*Revised 14 May 1998*
*Accepted 27 May 1998*

*Correspondence to*: F Levi, Registre Vaudois des Tumeurs, CHUV-Falaises 1, CH-1011 Lausanne, Switzerland

### MATERIALS AND METHODS

The data for the present report were abstracted from the Cancer Registry of the Canton of Vaud (Levi et al, 1997) whose population, according to the 1990 census, was about 600 000 inhabitants. The registry is tumour based, and multiple primaries occurring in the same patient are entered separately. The basic information available from the register comprises sociodemographic characteristics of the patient (i.e. age, sex), primary site and histological type of the tumour according to the standard International Classification of Diseases for Oncology (ICD-O; World Health Organization, 1976), and time of diagnostic confirmation. Passive and active follow-up are recorded.

Since 1976, a registration scheme, applying the same standardized rule as for incident malignancies, has been implemented for borderline ovarian tumours (Levi et al, 1993). Bilateral ovarian tumours (either synchronous or metachronous) are not registered separately, unless histologically different. Between 1976 and 1996, 176 histologically verified ovarian tumours labelled as of borderline malignancy or carcinomas of low malignant potential, according to the WHO International Classification of Tumours (Serov et al, 1973) and standard histological criteria and sampling technical requirements (Czernobilsky, 1987), were registered.

The vital status of each patient included in the present analysis has been actively verified up to 31 December 1996. On the basis of these data, survival rates were computed according to the product-limit (maximum likelihood) method. Relative survival was derived after allowance for the general life table of the canton. The persons included in the present series were also followed up to the end of 1996 for the occurrence of a second primary neoplasm, for a total of 1444 women–years at risk. Calculation of expected numbers of cases were based on site-, age- and calendar period-specific incidence rates, multiplied by the corresponding number of women–years at risk.

### RESULTS

A total of 176 borderline ovarian tumours were registered, corresponding to an age-adjusted overall incidence (world standard) of 2.7 per 100 000 in the 21-year calendar period considered. These represented 19% (176 out of 948) of all ovarian neoplasms. Incidence rose from 1.7 per 100 000 in 1976–81 to 2.7 in 1987–91, and then levelled off. Incidence rates tended to decline below age 55 (average change per year –8% at age 15–34 and –1.3% at age 35–54, although none of these estimates were significant), but increased in the elderly. One hundred and two (58%) cases were serous, 72 (41%) mucinous, and two (1%) other or unspecified. The age-specific incidence was 2.0 per 100 000 at age 15–34, rose to 4.1 per 100 000 at age 35–54, and to 4.3 and 4.7 at age 55–74 and ≥75 (Table 1).

Overall relative survival was 96% at 5 years, and 94% at 10 years. Ten-year survival was 96% in serous compared with 91% in mucinous neoplams (Figure 1).

A total of 18 second neoplasms were observed, compared with 10.3 expected. Of these, five (three of ovary and one of right colon and unknown origin respectively) were synchronous. There was a significant excess of invasive ovarian cancers (four observed, including three synchronous primaries, compared with 0.36 expected), but not of any other cancer site.

### DISCUSSION

The incidence rates of borderline ovarian tumours from this Swiss population are lower than those of the US or of Norway but higher than those of Finland or Israel, presumably in parallel with the difference in ovarian cancer incidence and mortality rates registered in these countries (Levi et al, 1994, 1998). This indirectly confirms that the determinants of invasive and borderline ovarian cancer are similar, at the population level (Parazzini et al, 1991 *a,b*).

The present series also confirms the clinical and epidemiological observation that the age curve for borderline ovarian tumours, after rising at younger age, peaks in the fourth and fifth decades, to level off thereafter, and that the prognosis of the disease is favourable, with 94% 10-year survival. This compares well with the 93% survival from US (Harlow et al, 1987) and the over 90% 10-year survival from Norway (Bjorge et al, 1998).

Of specific interest is the pattern of second neoplasms, showing a significant excess of invasive ovarian cancers in women with borderline neoplasms. This is partly attributable to increased diagnostic ascertainment, but may further suggest the existence of a common aetiological pathway between borderline and invasive ovarian cancer (Parazzini et al, 1991*a,b*; Leake et al, 1992).

The rises observed in incidence of borderline ovarian tumours up to the early 1990s, however, are not paralleled by invasive ovarian cancer trends in this population (Levi et al, 1996). This suggests that increased diagnostic attention and changes in the classification may at least in part explain observed rises, although new risk factors, such as treatments for fertility, have been associated with the risk of borderline ovarian cancers (Rossing et al, 1994; Parazzini et al, 1998). Incidence rates of borderline ovarian cancer, however, have been declining in women below age 55, and more notably below age 35, during the last decade, possibly reflecting a favourable impact of combined oral contraceptives not only on invasive (La Vecchia et al, 1996, 1998) but also on borderline ovarian tumours.

### ACKNOWLEDGEMENTS

The contribution of the Vaud Cancer Registry's staff, and the collaboration of the pathological laboratories operating in the Canton of Vaud, are gratefully acknowledged.

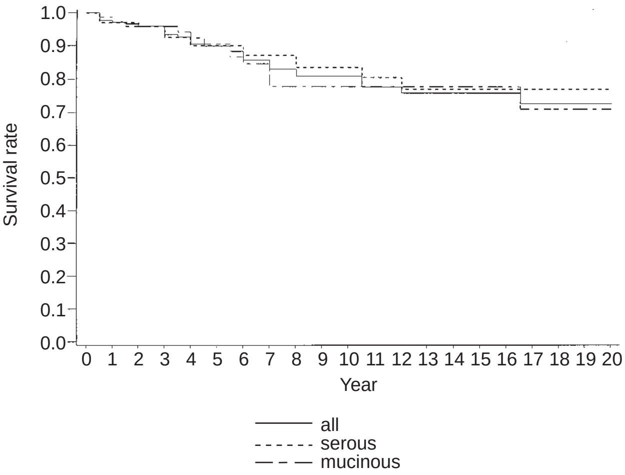

**Figure 1** Twenty-year survival curve for borderline ovarian tumours according to histological type. Vaud, Switzerland, 1976–96

**Table 1** Trends in age-standardized[a] incidence rates per 100 000 women for borderline ovarian tumours from the Vaud population, Switzerland, according to age group and calendar period, 1976–96

| Calendar period | Incidence rate per 100 000 at age | | | | |
|---|---|---|---|---|---|
| | 15–34 | 35–54 | 55–74 | ≥75 | All ages |
| | Rate (*n*) | Rate (*n*) | Rate (*n*) | Rate (*n*) | Rate (*n*) |
| 1976–81 | 0.51 (3) | 4.91 (20) | 2.93 (10) | 2.43 (3) | 1.72 (36) |
| 1982–86 | 3.02 (13) | 1.71 (6) | 3.25 (9) | 2.56 (3) | 1.80 (31) |
| 1987–91 | 2.61 (12) | 4.74 (19) | 5.14 (14) | 7.28 (9) | 2.71 (54) |
| 1992–96 | 2.25 (10) | 4.62 (20) | 5.99 (16) | 6.26 (9) | 2.66 (55) |
| 1976–96 | 2.03 (38) | 4.09 (65) | 4.29 (49) | 4.74 (24) | 2.22 (176) |
| Average change per year (%) | –8.5 | –1.3 | +4.9[b] | +7.3 | +3.2 |

[a]Age-standardized rates on the world population. [b]$P < 0.05$.

© Cancer Research Campaign 1999

### REFERENCES

Auranen A, Grénman S, Mäkinen J, Pukkala E, Sankila R and Salmi T (1996) Borderline ovarian tumors in Finland: epidemiology and familial occurrence. *Am J Epidemiol* **144**: 548–553

Barnhill D, Heller P, Brzozowski P, Advani H, Gallup D and Park R (1985) Epithelial ovarian carcinoma of low malignant potential. *Obstet Gynecol* **65**: 53–59

Bjorge T, Engeland A, Hansen S and Trope CG (1997) Trends in the incidence of ovarian cancer and borderline tumours in Norway, 1954–1993. *Int J Cancer* **71**: 780–786

Bjorge T, Engeland A, Hansen S and Trope CG (1998) Prognosis of patients with ovarian cancer and borderline tumours diagnosed in Norway between 1954 and 1993. *Int J Cancer* **75**: 663–670

Czernobilsky B (1987) Common epithelial tumors of the ovary. In *Blaustein's Pathology of the Female Genital Tract*. Kurman RJ (ed.), pp. 560–606. Springer-Verlag: New York

Harlow BL, Weiss NS and Lofton S (1987) Epidemiology of borderline ovarian tumors. *J Natl Cancer Inst* **78**: 71–74

Hart WR (1977) Ovarian epithelial tumors of borderline malignancy (carcinomas of low malignant potential). *Hum Pathol* **8**: 541–549

Iskovich J, Shushan A, Schenker JG and Paltiel O (1998) The incidence of borderline ovarian tumors in Israel. A population-based study. *Cancer* **82**: 147–151

Kærn J, Trope CG and Abeler VM (1993) A retrospective study of 370 borderline tumors of the ovary treated at the Norwegian Radium Hospital from 1970 to 1982. *Cancer* **71**: 1810–1820

Kennedy AW and Hart WR (1996) Ovarian papillary serous tumors of low malignant potential (serous borderline tumors). A long-term follow-up study, including patients with microinvasion, lymph node metastasis, and transformation to invasive serous carcinoma. *Cancer* **78**: 278–285

La Vecchia C, Tavani A, Franceschi S and Parazzini F (1996) Oral contraceptives and cancer. A review of the evidence. *Drug Safety* **14**: 260–272

La Vecchia C, Negri E, Levi F, Decarli A and Boyle P (1998) Cancer mortality in Europe: effects of age, cohort of birth and period of death. *Eur J Cancer* **34**: 118–141

Leake JF, Currie JL, Rosenshein NB and Woodruff JD (1992) Long-term follow-up of serous ovarian tumors of low malignant potential. *Gynecol Oncol* **47**: 150–158

Levi F, Franceschi S, La Vecchia C, Ruzicka J, Gloor E and Randimbison L (1993) Epidemiologic pathology of ovarian cancer from the Vaud Cancer Registry, Switzerland. *Ann Oncol* **4**: 289–294

Levi F, Lucchini F and La Vecchia C (1994) Worldwide patterns of cancer mortality, 1985–89. *Eur J Cancer Prev* **3**: 109–143

Levi F, Te VC, Randimbison L and La Vecchia C (1996) Trends in cancer incidence and mortality in Vaud, Switzerland, 1974–1993. *Ann Oncol* **7**: 497–504

Levi F, Te VC, Randimbison L and La Vecchia C (1997) Statistics from the Registry of the Canton of Vaud, Switzerland, 1988–1992. In *Cancer Incidence in Five Continents*, vol VII. Parkin DM, Whelan S, Ferlay J, Raymond L and Young J (eds), pp. 674–677. IARC Scientific Publication 143: Lyon

Levi F, Lucchini F, Boyle P, Negri E and La Vecchia C (1998) Cancer incidence and mortality in Europe, 1988–92. *J Epidemiol Biostat* **3**: 295–373

Nakashima N, Nagasaka T, Oiwa N, Nara Y, Fukata S, Fukatsu T and Takeuchi J (1990) Ovarian epithelial tumors of borderline malignancy in Japan. *Gynecol Oncol* **38**: 90–98

Parazzini F, Franceschi S, La Vecchia C and Fasoli M (1991*a*) The epidemiology of ovarian cancer. *Gynecol Oncol* **43**: 9–23

Parazzini F, Restelli C, La Vecchia C, Negri E, Chiari S, Maggi R and Mangioni C (1991*b*) Risk factors for epithelial ovarian tumours of borderline malignancy. *Int J Epidemiol* **20**: 871–877

Parazzini F, Negri E, La Vecchia C, Moroni S, Polatti A, Chiaffarino F, Surace M and Ricci E (1998) Treatment for fertility and risk of ovarian tumors of borderline malignancy. *Gynecol Oncol* **68**: 226–228

Rice LW, Berkowitz RS, Mark SD, Yavner DL and Lage JM (1990) Epithelial ovarian tumors of borderline malignancy. *Gynecol Oncol* **39**: 195–198

Rossing MA, Daling JR, Weiss NS, Moore DE and Self SG (1994) Ovarian tumors in a cohort of infertile women. *N Engl J Med* **331**: 771–776

Scully RE (1982) Common epithelial tumors of borderline malignancy (carcinomas of low malignant potential). *Bull Cancer* (Paris) **69**: 228–238

Serov SF, Scully RE and Sobin LH (1973) Histological typing of ovarian tumours. In *International Histological Classification of Tumours*. No. 9, pp. 17–18 and p 37. World Health Organization: Geneva

World Health Organization (1976) *International Classification of Diseases for Oncology (ICD-O)*, p 131. World Health Organization: Geneva

*© Cancer Research Campaign 1999*
